# Symptom Trajectory and Readmission Risk Prediction in Chronic Respiratory Failure via Latent Class Growth Model

**DOI:** 10.1093/geroni/igaf122.1616

**Published:** 2025-12-31

**Authors:** Rong Zhang

**Affiliations:** Shanxi Bethune Hospital, Taiyuan, Shanxi, China (People’s Republic)

## Abstract

**Objective:**

This study employs the Latent Class Growth Model(LCGM) to identify symptom trajectory categories in patients with chronic respiratory failure(CRF)and examines the association between distinct trajectory categories and readmission risk.

**Methods:**

A retrospective cohort study was conducted, encompassing CRF patients hospitalized at a tertiary comprehensive hospital between January 2022 and December 2024.Data on demographic characteristics, clinical indicators, symptom scores, and readmission rates were collected.LCGM was applied to model symptom trajectories and identify latent trajectory categories.Additionally, multivariate Cox proportional hazards models were utilized to examine the relationship between different trajectory categories and readmission risk.

**Results:**

A total of 512 CRF patients were included.LCGM analysis identified three distinct symptom trajectory categories: stable, fluctuating, and deteriorating. Patients in the stable category exhibited minimal variations in symptom scores over time, while those in the fluctuating category demonstrated periodic fluctuations.Conversely, patients in the deteriorating category experienced a continuous escalation in symptom scores. Multivariate Cox regression analysis revealed that, compared to the stable trajectory, patients classified under the fluctuating and deteriorating categories exhibited significantly increased readmission risks, with hazard ratios of 1.56 and 2.34, respectively. Moreover, patients in the deteriorating category were found to have substantially longer hospital stays and significantly higher medical expenditures than those in the other two categories.

**Conclusion:**

This study successfully identified three distinct symptom trajectory categories in CRF patients through the application of LCGM and established that fluctuating and deteriorating symptom patterns were significantly correlated with an increased risk of readmission. The findings highlight the necessity of prioritizing clinical attention toward patients exhibiting fluctuating and deteriorating symptom trajectories, implementing early interventions, and adopting personalized management strategies to mitigate readmission rates and enhance patient outcomes.

